# Evidence for a radiation-responsive ‘p53 gateway’ contributing significantly to the radioresistance of lepidopteran insect cells

**DOI:** 10.1038/s41598-017-18521-5

**Published:** 2018-01-08

**Authors:** Ashish Kumar, Sudhir Chandna

**Affiliations:** 1 Department of Genetics, Texas Biomedical Research Institute, 7620 NW Loop 410, San Antonio, Texas 78227 USA; 2 Division of Natural Radiation Response Mechanisms, Institute of Nuclear Medicine & Allied Sciences, Brig. S.K. Mazumdar Road, Timarpur, Delhi 110054 India

## Abstract

Recently, we have demonstrated that microRNA-31 (miR-31) overexpression is inherent to radiation-induced cell death in the highly radioresistant Sf9 insect cells, and regulates pro-apoptotic Bax translocation to mitochondria. In the present study, we report that at sub-lethal radiation doses for Sf9 cells, miR-31 is significantly downregulated and is tightly regulated by an unusual mechanism involving p53. While ectopic overexpression of a well-conserved Sfp53 caused typical apoptosis, radiation-induced p53 accumulation observed selectively at sub-lethal doses failed to induce cell death. Further investigation of this paradoxical response revealed an intriguing phenomenon that sub-lethal radiation doses result in accumulation of a ‘hyper-phosphorylated’ Sfp53, which in turn binds to miR-31 genomic location and suppresses its expression to prevent cell death. Interestingly, priming cells with sub-lethal doses even prevented the apoptosis induced by lethal radiation or ectopic Sfp53 overexpression. On the other hand, silencing p53 increased radiation-induced cell death by inhibiting miR-31 downregulation. This study thus shows the existence of a unique radiation-responsive ‘p53 gateway’ preventing miR-31-mediated apoptosis in Sf9 cells. Since Sfp53 has a good functional homology with human p53, this study may have significant implications for effectively modulating the mammalian cell radioresistance.

## Introduction

Ionizing radiation (IR) leads to double-strand DNA breaks or DSBs which activate cell-cycle checkpoints to initiate a cohort of signals ultimately leading to determination of cell fate such as cell death, damage free cell survival or even cellular transformation. Tumor suppressor p53 is one of the most extensively studied DNA damage responsive proteins, which regulates cellular radiation response and is also known to be frequently mutated in human tumors. Signaling network of p53 involves hundreds of genes and proteins that play important role in maintaining genomic stability, tumor suppression as well as in cellular responses to various types of genotoxic insults^1,2^. Following exposure to ionizing radiation or other DNA damaging agents, the level of intracellular p53 increases primarily via inhibited degradation, and is associated with nuclear translocation and increased transcriptional activity. Accumulation of p53 in the nucleus activates a variety of downstream signaling pathways including cell cycle checkpoints that facilitate DNA repair, or alternatively the intrinsic pathway of apoptosis when damage is irreparable. It is also well documented that certain mutations in TP53 gene can lead to increased radioresistance mainly either by transactivating DNA repair genes or by altering G1 cell cycle arrest, whereas wild type P53 has been shown to be associated with radiosensitivity in a variety of tissues^3–7^.

Recent studies have also revealed close interaction between p53 and certain miRNAs. Stress induced accumulation/activation of p53 is shown to regulate the expression of various miRNAs both at transcriptional and post-transcriptional levels^8–10^. For example, p53-mediated upregulation of miR-34 is known to induce cell death in *C*. *elegans* as well as in mammalian cells^11,12^. Many other miRNAs other than miR-34 family members are now known to be regulated by p53, viz., miR-194, miR-207, miR-107^13^, miR-215, miR-192^14,15^ miR-16-1, miR-143, miR-145, and miR-216^9^. Mutations in p53 are shown to promote cancer progression by altering the expression of certain miRNAs^16^. On the other hand, certain miRNAs may also regulate the expression and/or function, either directly by negative regulation of p53 protein (miR-504^17^, miR-125b^18^) or indirectly (by miR-34a, miR-29 and miR-122, reviewed by Feng Z. *et* *al*.^8^) through regulation of p53 regulators. Many more miRNAs that may be directly or indirectly regulated by p53 remain to be identified, and some of these miRNAs may also be important in the radiation-induced cell death regulation.

A recent study from our laboratory has demonstrated mediatory role of miR-31 in radiation-induced cell death in a radioresistant insect cell line (Sf9) that carries numerous structural and functional similarities with mammalian/human cells^19^. Radiation-induced overexpression of miR-31 was shown to promote Bim/Bax-mediated apoptosis in these cells at lethal doses of ionizing radiation. In contrast, downregulation of miR-31 was observed at sub-lethal doses, a response that could be associated with radiation resistance in this model system. MiR-31 has also been reported to alter the radiosensitivity of esophageal adenocarcinoma *in vitro* ^20^. Interestingly, contradicting data from different studies have shown miR-31 as tumor suppressor/oncogenic miRNA and indicated that its role in carcinogenesis may be cell type dependent^21,22^. An isolated study reported that miR-31 is the most strongly downregulated miRNA in serous ovarian tumors as well as in many osteosarcoma and pancreatic carcinoma cell lines. Functional analysis strongly indicated that tumor-suppressive miR-31 inhibits proliferation and promotes apoptosis in the ovarian tumor cells. Interestingly, these effects could be observed only in cell lines with a dysfunctional p53 signaling pathway^23^.

The existence of p53 homologue in Sf9 cells has been reported earlier^24,25^ and its functional characterization has shown altered radiation response as compared to human p53^26,27^. We have previously shown comparison of Sfp53 responses with mammalian cells, at radiation doses relevant to mammalian cells^24^. However, some studies suggested the response of Sfp53 following DNA damaging baculovirus infection^27,28^, the basis of this altered ‘radiation response’ of Sfp53 and its role in radiation-induced cell death has not been established clearly. Since Sf9 cells also have an active miR-31 response during radiation-induced cell death^19^, we conducted the present study for exploring possible relationship between Sfp53 and miR-31 response following radiation exposure. The study shows a rather interesting role of p53 phosphorylation status in the radioresistance of Sf9 cells, which seems to work through highly selective suppression of miR-31 expression at sub lethal doses.

## Results

### Radiation-induced accumulation and nuclear translocation of Sfp53 corresponds inversely with the alterations in miR-31 expression

**Figure 1:**
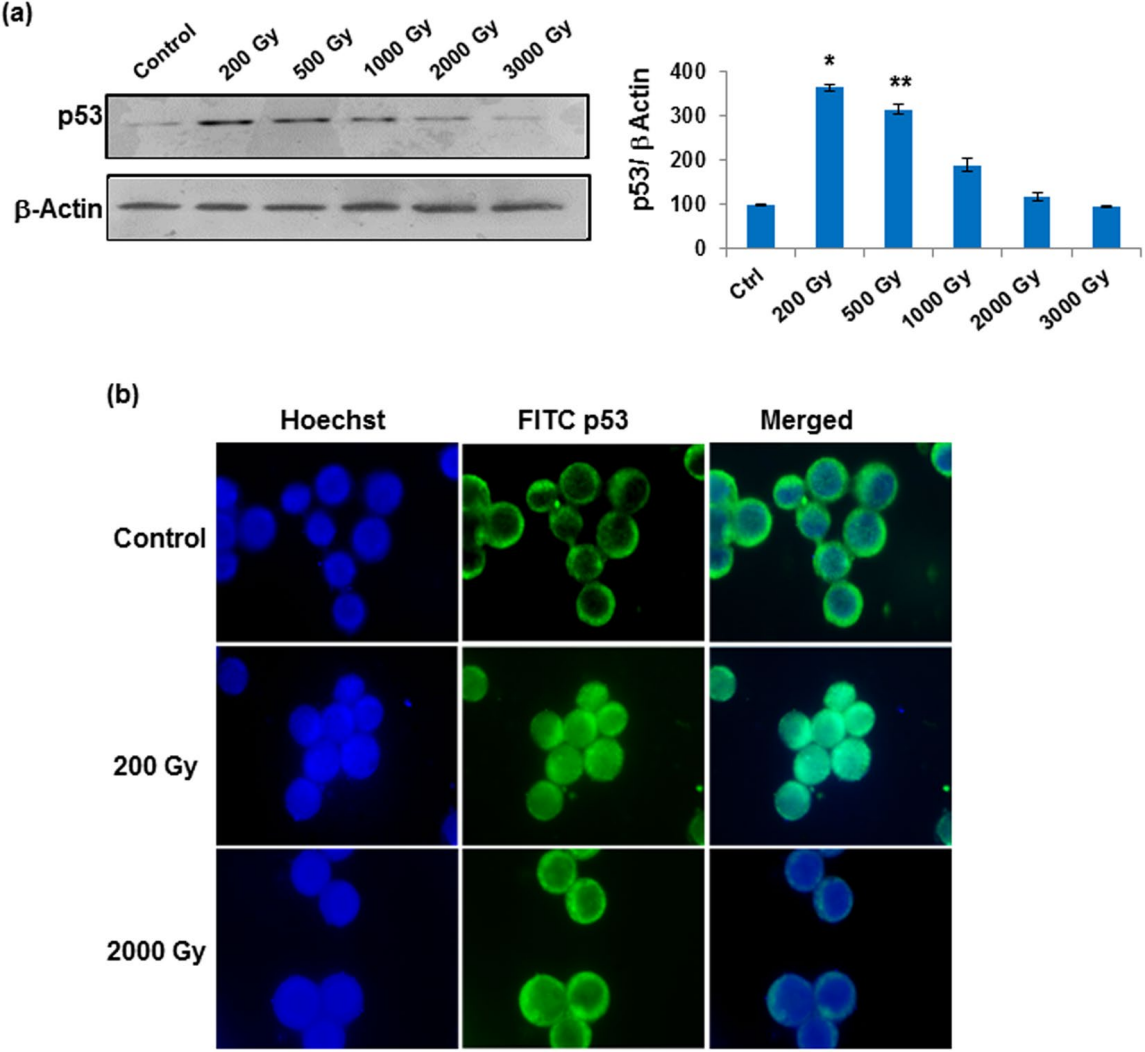
Radiation induced accumulation and translocation of Sfp53 to the nucleus is not associated with miR-31 dependent cell death. (**a**) Western blot of Sfp53 following varied doses of g-radiation at 24 h time point showed its accumulation at sub-lethal doses. Densitometry analysis was used to quantitate the western blots. (**b**) Following irradiation with sub-lethal and lethal doses Sfp53, immuno-labelled with anti-p53 FITC antibody, showed its nuclear translocation selectively at sub-lethal doses. Nucleus was counterstained with Hoechst. Images were captured at 40X magnification. Images are representative of three independent experiments.

Irradiating radioresistant Sf9 cells with varying doses ranging up to 3 kGy caused significant accumulation and nuclear translocation of Sfp53, but this phenomenon was most prominent at relatively lower doses (<500 Gy) that failed to induce cell death. At doses 500 Gy-3 kGy, Sfp53 accumulation reduced progressively in a dose-dependent manner, and its level at 2 kGy-3 kGy was comparable with that of untreated control. The p53 translocation to nucleus was also prominent at the sub-lethal dose of 200 Gy, while only partial translocation could be observed at lethal doses (Fig. 1b).

Importantly, radiation-induced accumulation/expression of Sfp53 showed inverse relationship with the alterations in expression of miR-31, which was recently shown to regulate caspase activation and apoptotic induction in these cells^19^. While p53 showed maximal accumulation following irradiation at 200 Gy (Fig. 1a), miR-31 expression was previously reported to be unusually suppressed at this dose that failed to induce caspase-3 activity or cell death^19^. On the other hand, irradiation at 1 kGy–3 kGy was also shown to increase caspase-3 activity and cell death in dose dependent manner. These data thus show an inverse relationship between radiation-induced p53 accumulation and miR-31 expression, the overexpression of latter coinciding with cell death induction as reported earlier^19^.

### Sfp53 failed to regulate the canonical p53 transcriptional targets despite radiation-induced accumulation and nuclear translocation

**Figure 2:**
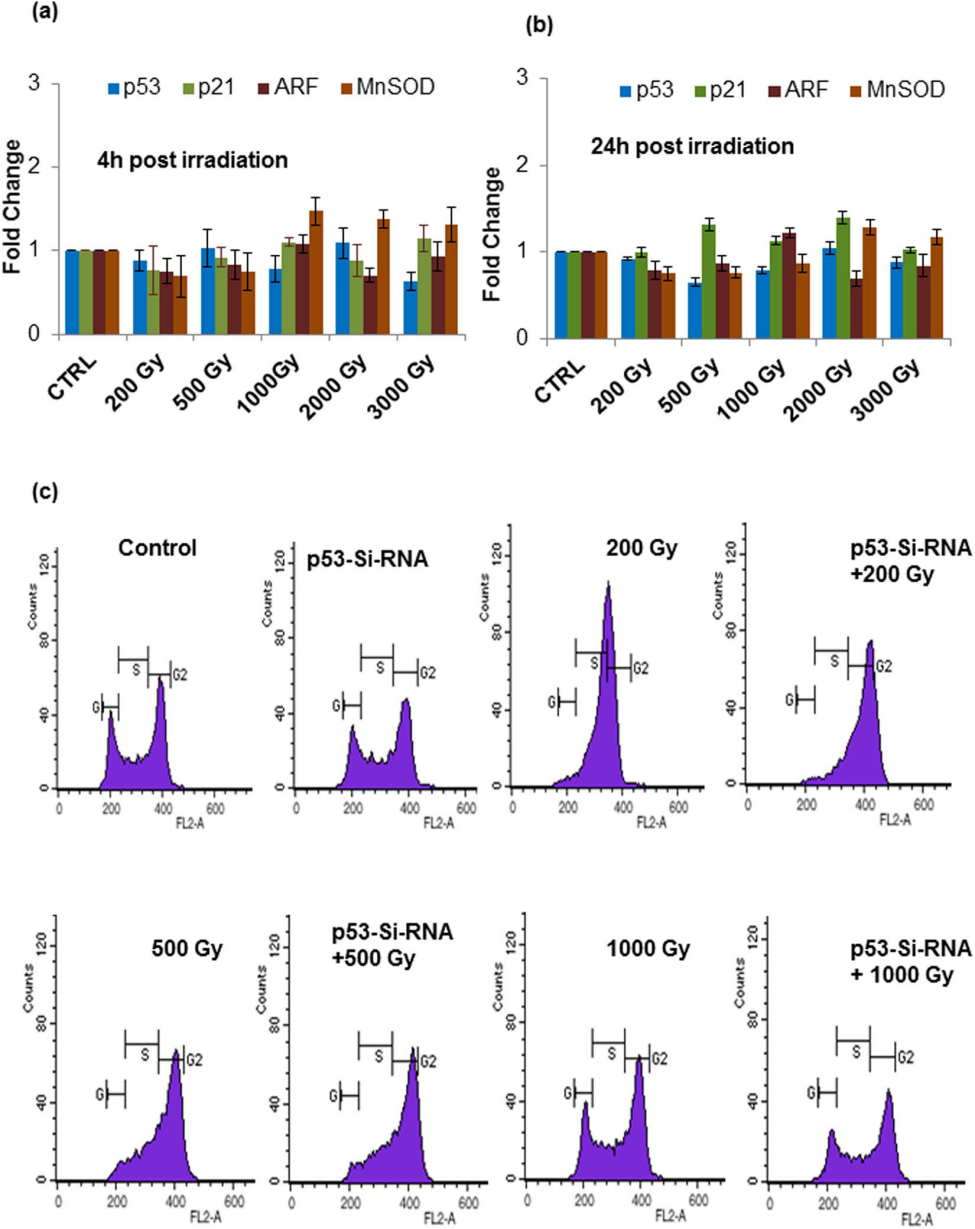
Sfp53 is incompetent of regulating cell cycle perturbations and expression of its transcriptional targets following irradiation. (**a**,**b**) Real time PCR on some of the canonical transcriptional targets of p53 showed its transcriptional inactivity after irradiation at early (4 h) and late (24 h) time points. The fold change values for MnSOD ranged from 0.8 to 1.45 (p = 0.103) and p21 fold change values ranged 0.9 to 1.4 (p = 0.076). (**c**) Cell cycle distribution using flow cytometry was performed on harvested cells 24 h post irradiation with or without siRNA against p53. No change in cell cycle distribution was observed with p53 siRNA indicating no participation of p53 in the regulation of cell cycle perturbation. Data are mean ± SD of three independent experiments.

In agreement with the known human p53 response, Sfp53-mRNA levels remained unchanged following irradiation (Fig. 2a,b). Therefore, Sfp53 protein seems to ‘accumulate’ in response to radiation. This indicated that Sfp53 may have similar functional activity following radiation stress as is classically established for human p53. However, the radiation-induced accumulation of Sfp53 at sublethal doses was not found to be linked with the canonical transcriptional activity. Some of the known p53 target genes, viz., p21, Mn-SOD and ARF, were used to investigate transcriptional activity of Sfp53 by assessing alterations in their post-irradiation mRNA expression levels, which remained either unchanged or were slightly increased (non-significant ‘p’ values) at early (4–6 h) as well as late (24 h) time-points (Fig. 2a,b). Moreover, silencing Sfp53 using siRNA did not show any effect on the radiation-induced cell cycle perturbation (Fig. 2c). These observations strongly suggest that Sfp53 is not able to regulate these transcriptional targets, and may not be playing similar role in radiation-induced cell cycle checkpoints or cell death regulation as generally observed in human cells.

### *In silico* characterization of Sfp53 suggests well-conserved functional integrity

**Figure 3:**
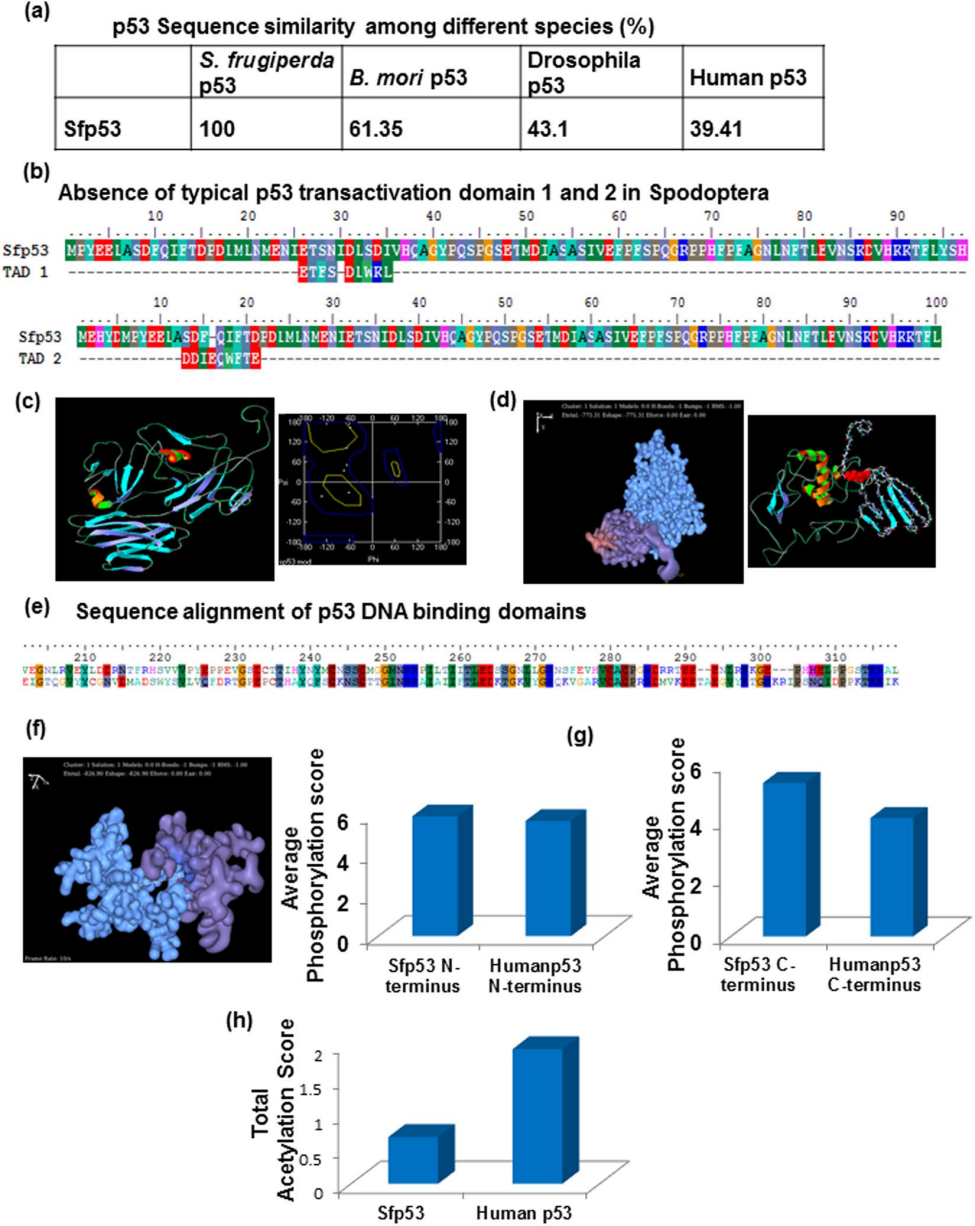
*In-Silico* analysis point towards functional integrity of Sfp53, despite having structural dissimilarities with human/*B*. *mori*/*Drosophila* p53. (**a**) Sequence alignment of Sfp53 with human/*B*.*mori*/*Drosophila* p53 showed no considerable similarities. Also (**b**) Sfp53 did not possess typical 9 amino acid transactivation domain 1 & 2. (**c**) Protein sequence of Sfp53 was used for the *ab-initio* modelling using I-TASSER online tool and the protein model (Left panel) was verified by engendering Ramchandran plot (right panel). (**d**) N-terminus of modelled Sfp53 (blue solid dots model) showed possible interaction with lepidopteran TAF9 (purple solid dots model). Ribbon model on the right panel showed the interaction site (red solid dots) which corresponds to the amino acids 17–25 of Sfp53. (**e**) DNA binding domain of Sfp53 was observed to have perfect sequence conservation with human p53 for the amino acids known to interact directly with DNA. Further (**f**) dimerization potential of Sfp53 was analyzed by docking two individual subunits of the C-terminus of Sfp53. (**g**) The N and C terminus of p53 from S. *frugiperda* and human were analyzed for their phosphorylation score. Selectively C-terminus of Sfp53 was predicted to be hyper-phosphorylated. Also (**h**) Sfp53 was predicted to be in hypo-acetylated state.

For *in silico* characterization of Sfp53, the protein sequence of *Spodoptera frugiperda* p53 was extracted from NCBI database (AEC04309.1). BLAST analysis of Sfp53 with *Homo sapiens* p53 showed only 39.41% similarity and 24.33% identity. Importantly, Sfp53 also failed to show considerable similarity either with *Bombyx mori* p53 (bmp53; 61.35%) or *Drosophila melanogaster* p53 (Dmp53; 43.1%) (Fig. 3a). Earlier, it has been suggested that Sfp53 shares good level of functional similarity with *Drosophila* p53 with respect to transactivation, DNA binding nuclear localization, and oligomerization despite having significant dissimilarities between their protein sequences^25^. Sfp53 has also been found to be deficient in both the typical nine amino acids long transactivation domain (Fig. 3b). The primary sequence of Sfp53 has further been used for structural modeling using I-TASSER online tool^29^. We further analyzed the reliability of modeled structure by generating Ramachandran Plot (Fig. 3c). In order to confirm the functional transcriptional activity of Sfp53, the N-terminus of modeled Sfp53 was selected to analyze its interaction with lepidopteran (*B*. *mori*) TAF9 (XP_004923952.1) using the Hex 6.2 software^30^. Results strongly suggest a positive interaction of N-terminus of Sfp53 with TAF9 (Fig. 3d). Amino acids 17–25 of Sfp53 N-terminus were found to be in close proximity with the TAF9 and depict possible interaction site, which is in line with the previous observations suggesting common interaction site (amino acids 17–26) of TAF9 and MDM-2 with p53 in the mammalian system^31,32^. These observations thus suggest that Sfp53 can interact with other transcriptional factors despite lacking typical nine amino acids transactivation domains, and is hence likely to have functionally active transcriptional domain.

Further, to test the conservation of DNA binding domain between Sfp53 and human p53, sequence alignment of this region was performed. The DNA binding domain of both the p53 orthologues was found to be nearly identical in terms of conservation of amino acids as well as the positions of amino acids interacting with DNA (Fig. 3e). This observation is in line with the previous report from our group which showed that Sfp53 can bind perfectly on the human MDM-2 promoter site^24^.

Dimerization ability of Sfp53 was also observed using Hex 8.0 software. The C-terminus of Sfp53 in its original conformation (as in full length Sfp53 model) was used and its interaction with another monomer of Sfp53 C-terminus was studied. Positive interaction of two C-termini with good ‘E’ total (−826.90) was evident in this analysis (Fig. 3f). Further, phosphorylation scores of N- and C- termini of Sfp53 were obtained. Interestingly, the C-terminus of Sfp53 is predicted to have significantly higher phosphorylation score than the human p53 (Fig. 3g). Also, average acetylation score of Sfp53 was found to be significantly less than human p53 which suggests that radiation-induced post-transcriptional modifications of Sfp53 may be responsible for its inactivation^33–35^. The lower acetylation score of Sfp53 (Fig. 3h) was also well corroborated by the previously reported relatively higher (≈70%) HDAC activity in these cells in comparison to mammalian cells^36^. In summary, the entire *in silico* analysis pointed towards a well-conserved Sfp53 radiation response, except for the post-translational modification scores being significantly different from hp53.

### Ectopic expression of Sfp53 induced apoptotic cell death in Sf9 cells

**Figure 4:**
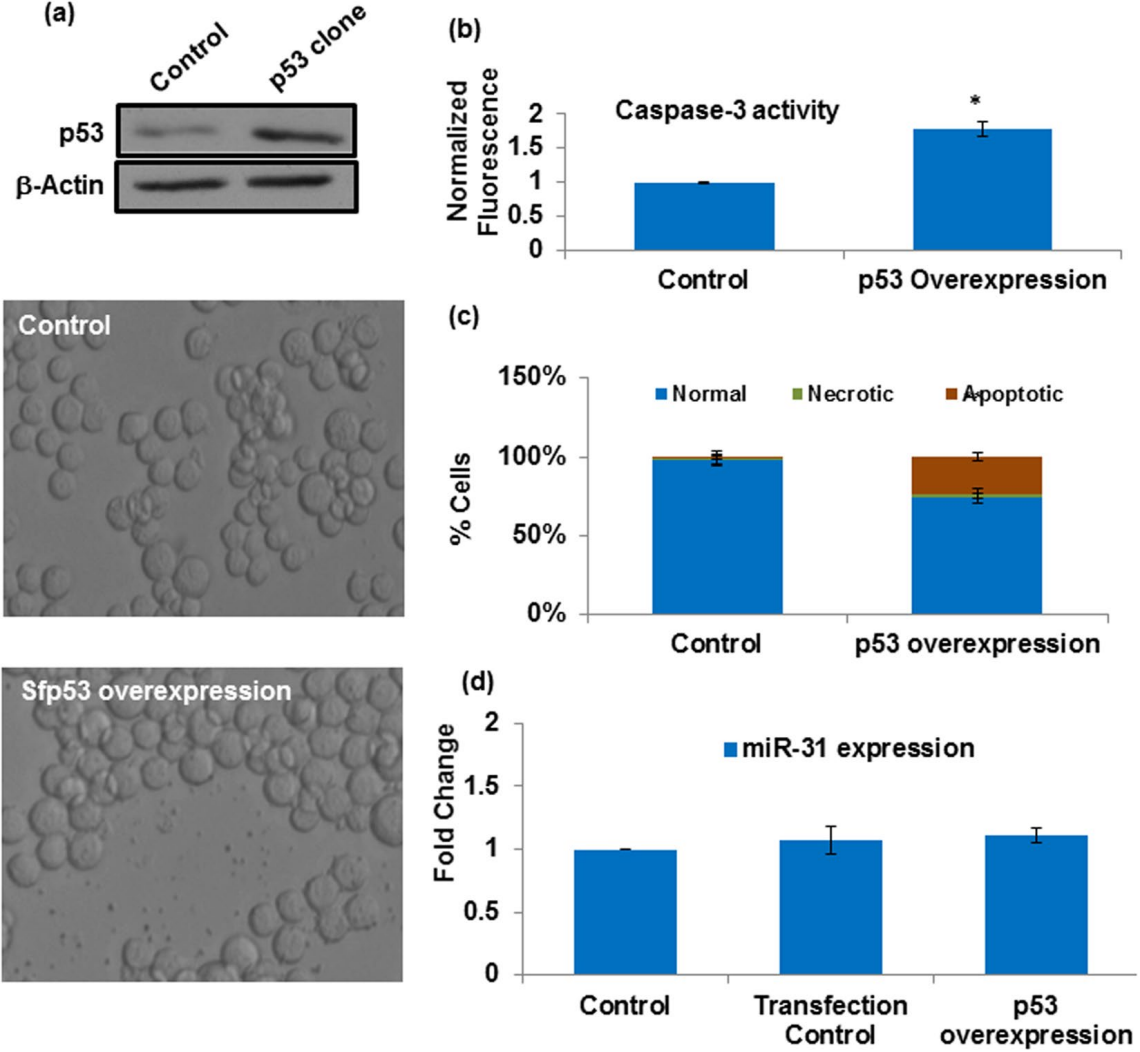
Ectopic overexpression of Sfp53 induces caspase-3 dependent apoptosis in miR-31 independent manner. (**a**) Sf9 cells were harvested 24 h following transfection with Sfp53 clone. Significant cell death was observed under DIC microscope. Cells were then harvested and immuno-blotted with p53 specific antibody. Cell death/apoptosis was also measured 24 h post transfection by (**b**) caspase-3 activation and (**c**) morphological analysis. Data are mean ± SD of three independent experiments. (*P < 0.001, **P < 0.02) (**d**) Sf-miR-31 showed no change in expression, measured by q-PCR 24 h after Sfp53 clone transfection.

*In silico* characterization of Sfp53 strongly indicated that this protein may be functionally active in native conformation. In order to validate the *in silico* observations, full length Sfp53 gene was cloned and overexpressed in Sf9 cells. Overexpression of Sfp53 alone, in the absence of any treatment, was observed to induce apoptotic cell death which initiated by 24 h post-transfection. (Fig. 4a–c). This observation suggests a functional role of Sfp53, and is in line with the previous study that indicated this as a functionally active p53 homologue^25^. It is further important to note that over-expression of Sfp53 also failed to alter miR-31 expression (Fig. 4d), which indicates that cell death induced by Sfp53 in this case might be executed through different mechanisms than the apoptosis induced by lethal radiation doses. Moreover, this pro-apoptotic activity of over-expressed p53 was paradoxically opposite to the lack of cell death observed in the case of p53 accumulation induced by the sub-lethal (200 Gy–500 Gy) radiation doses. Interestingly, the results also indicate that Sfp53 overexpression in the absence of irradiation is insufficient to inhibit miR-31 expression (Fig. 4d). We thus explored the basis of such contrasting effects of p53 overexpression versus radiation-induced accumulation, as detailed below.

### Sub-lethal radiation causes hyper-phosphorylation of Sfp53

**Figure 5:**
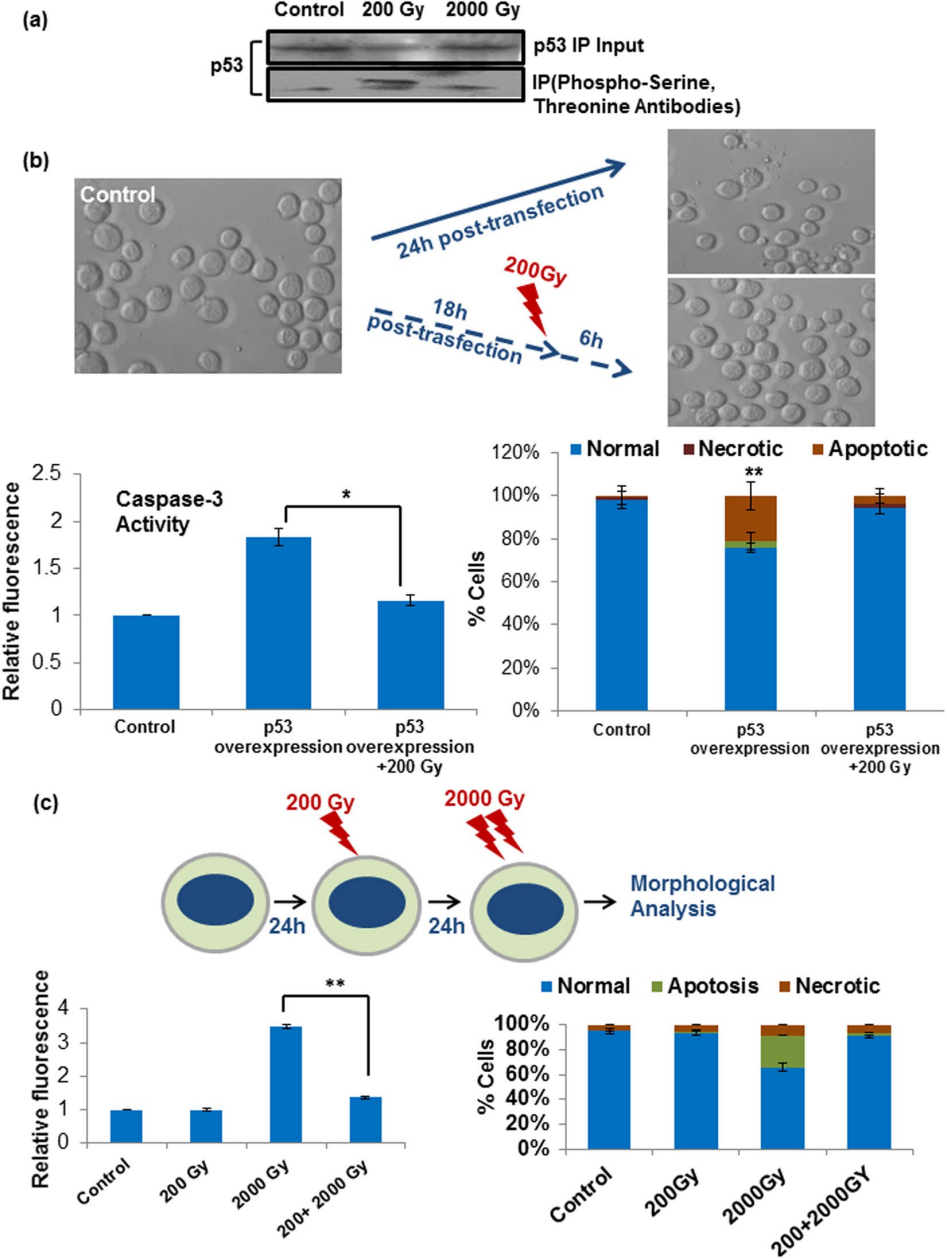
Low dose radiation cause hyper-phosphorylation of Sfp53 and protects Sf9 cells against radiation induced apoptosis. (**a**) Sf9 cells were harvested and lysed following irradiation with lethal and sub-lethal doses. Sfp53 was immuno-precipitated using p53 specific antibody and resolved on PAGE to detect its radiation induced phosphorylation at different doses using phosphor-serine/threonine antibodies. Hyper-phosphorylation of Sfp53 was observed following irradiation with sub-lethal doses. (**b**) To test the cyto-protective of phosphor-Sfp53, cells were irradiated with 200 Gy radiation dose 16 h post transfection to phosphorylate newly translated Sfp53. Cell death was measured by caspase-3 activation and morphological analysis after total 24 h post transfection between irradiated v/s non-irradiated transfected cells. Cell death was found to be significantly reduced by irradiating cells with 200 Gy after p53 clone transfection, showing its cyto-protective role. Data are mean ± SD of three independent experiments. (*P < 0.001, **P < 0.05). (**c**) Further, Sf9 cells were primed with 200 Gy (to simulate the condition of phosphorylated p53 overexpression) and then encountering the cells with lethal dose of radiation. The effect of priming the cells with low dose of radiation was measured in terms of cell death by caspase-3 activation and morphological analysis. Data are mean ± SD of three independent experiments. (*P < 0.002).

Since the activity of Sfp53 was found diametrically opposite in the two varying conditions of p53 accumulation or overexpression, we further assessed the level of phosphorylation in the irradiated versus unirradiated cells. Results show a very important and interesting anomaly, with a drastically higher level of phosphorylation selectively in the 200 Gy-irradiated cells than either the lethally irradiated (2000 Gy) or the unirradiated cells (Fig. 5a). Incidentally, 200 Gy dose induced maximum accumulation of p53 (Fig. 1a) and failed to induce cell death. In order to further confirm this newfound and intriguing cytoprotective role of hyper-phosphorylated Sfp53, we conducted dual treatment experiments involving Sfp53 over-expression later followed by irradiation at the sub-lethal dose of 200 Gy. Very importantly, the radiation-induced phosphorylation of over-expressed (and hence potentially lethal) p53 caused near-complete inhibition of caspase-3 activity and apoptosis (Fig. 5b).

Further to confirm whether 200 Gy-induced hyper-phosphorylation of Sfp53 plays cyto-protective role against subsequent insults, this sub-lethal dose was used for priming of the cells 16h–20h before irradiation with the lethal dose of 2000 Gy (Fig. 5c). Quite interestingly again, the induction of apoptosis by 2000 Gy was found considerably inhibited by preceding it with a priming 200 Gy dose (Fig. 5c). Therefore, these experiments confirm that phosphorylation of Sfp53 induced selectively at sublethal radiation doses has a considerable cyto-protective role. Possible downstream mechanisms through which the richly phosphorylated p53 may prevent cell death needed further investigations, as detailed in the next section.

### Newfound relation between radiation-responsive p53 and miR-31: hyper-phosphorylated Sfp53 binds near miR-31 locus and suppresses its expression to prevent apoptosis

**Figure 6:**
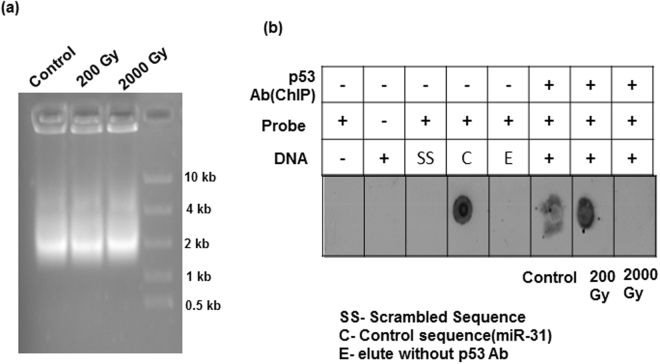
Phospho-Sfp53 binds nearby to Sf-miR-31 locus and selectively repress its expression at sub-lethal doses. (**a**) After DNA crosslinking, Sf9 cells were extracted and subjected to sonication to achieve DNA fragment size of 1000–2000 bp following irradiation with different doses. Immunoprecipitation of the fragmented DNA was performed using p53 antibody. In the immuno-precipitated DNA fragments, the presence of miR-31 sequence was identified using (**b**) dot blot analysis, taking miR-31 specific biotinylated probes to show p53 binding near to (1000–2000 bp region) miR-31 sequence. Results suggests Sfp53 binding selectively only at 200 Gy radiation, while in control condition moderate binding of Sfp53 near to miR-31 sequence was observed. Images are representative of three independent experiments.

Initial results during this study indicated that radiation-induced alterations in miR-31 expression have an inverse dose response with those in Sfp53 expression (Fig. 1a,c). In order to assess whether there is cause-and-consequence relationship between p53 and miR-31 expression and alterations following irradiation, direct binding of p53 near the miR-31 locus was tested using chromatin immunoprecipitation (ChIP) assay. While designing this strategy, long flanking sequences around SfmiR-31 were used since the exact location of p53 binding at miR-31 promoter site is not possible to predict in Sf9 cells due to non-availability of its annotated genome sequence. Binding of p53 around the ~1000–2000 bp region of miR-31 sequence was assessed through ChIP strategy in untreated Sf9 cells as well as cells that were irradiated with sublethal or lethal doses. Results from this ChIP assay combined with dot blot using biotinylated probes against miR-31 clearly show that p53 binds near the genomic miR-31 sequence site, albeit under selective conditions, viz., following its hyper-phosphorylation induced by 200 Gy dose (Fig. 6b). On the other hand, binding of p53 near miR-31 locus was not observed at the lethal dose of 2000Gy. This differential or selective binding of p53 at miR-31 locus following sub-lethal irradiation suggests a possible regulation of miR-31 expression by Sfp53 when the latter is in hyper-phosphorylated state. Moreover, the absence of miR-31 binding site at the 3′UTR of Sfp53 suggests that the Sfp53 expression is not influenced by miR-31 (Data not shown).

### RNA-based silencing of Sfp53 following sublethal dose irradiation enhances cell death associated with release of miR-31 suppression

**Figure 7:**
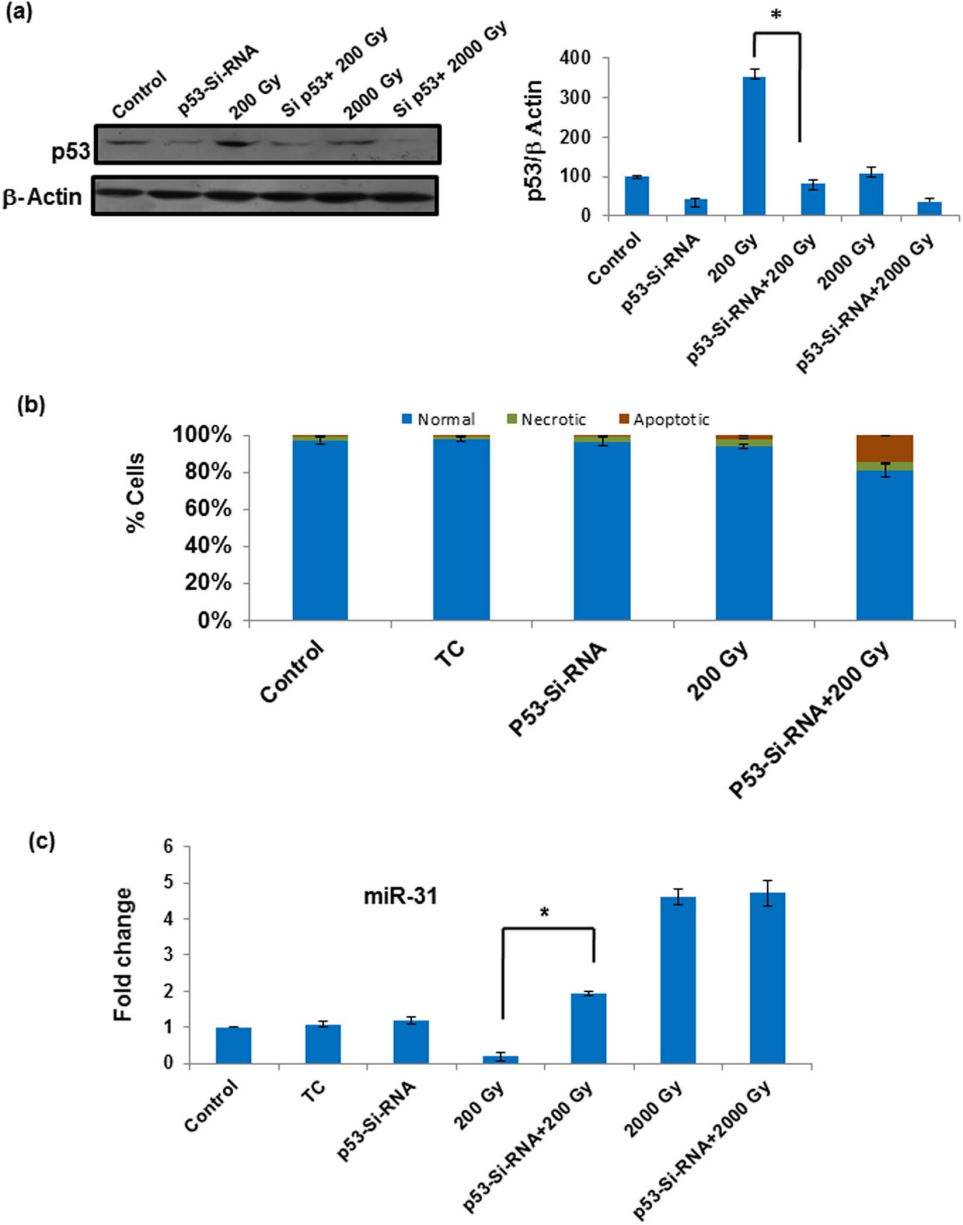
p53-si-RNA induce cell death at sub-lethal doses by inhibiting miR-31 downregulation. (**a**) Sf9 cells were transfected with Sfp53-siRNA 24 h before irradiating the cells with different doses. Cells were harvested and lyse for western blot analysis of Sfp53 24 h post irradiation. Sfp53 expression was quantitated from western blot using densitometry. (**b**) After Sfp53-siRNA transfection and irradiation, cell death was measured by Caspase-3 activation assay which showed significant cell death at sub-lethal doses. (**c**) Also the expression of miR-31 was found to significantly increase by inhibiting Sfp53, while at higher doses no change in miR-31 expression was observed. Data are mean ± SD of three independent experiments. (*P < 0.002).

To finally confirm whether radiation-induced accumulation of Sfp53 at sublethal doses plays any role in cytoprotection against radiation-induced apoptosis and/or downregulation of miR-31, Sfp53 expression was silenced using siRNA starting nearly 4 h before irradiation at the sublethal dose of 200 Gy (Fig. 7a). Accumulation of Sfp53 (usually observed 24 h post-irradiation) was found to be compromised by about 60% in the presence of siRNA, and resulted in considerable cell death (20%) even at this sub-lethal dose (Fig. 7b). Most importantly, this was also associated with nearly complete reversal of miR-31 repression induced by this sub-lethal dose (Fig. 7c), hence corroborating the presence of a selective radiation-induced Sfp53 gateway withholding miR-31 overexpression and cell death.

## Discussion

This study presents a novel radioprotective role of lepidopteran p53, a phenomenon hitherto unknown in any higher eukaryotic organism. Incidentally, the lepidopteran insects/insect cells display an unusual level of radioresistance, which makes this study even more important. Our findings show that Sfp53 negatively regulates the radiation-inducible overexpression of pro-apoptotic miR-31 in the highly radioresistant Sf9 cells reported recently by our laboratory^19^. While over-expression of miR-31 was prominent at the higher (lethal) doses that failed to accumulate p53, silencing p53 expression also resulted in revival of miR-31 expression associated with significant cell death induction, thereby confirming the existence of a ‘radioprotective p53 gateway’ in these model radioresistant cells. These findings carry substantial significance since Sfp53 does not have any major structural differences from its mammalian orthologues, and carries very good functional homology with human p53 (Fig. 3). *In silico* characterization such as sequence alignment, *ab initio* modeling, as well as interaction studies (using Hex 6.2 and 8.0), confirm a good level of functional conservation between the human p53 and Sfp53, and predict latter’s interaction with the transcriptional factor TAF9 that is required for transactivation of p53’s canonical targets (Fig. 2).

A very well conserved lLepidopteran p53, however, failed to respond with a typical dose-dependent radiation-induced accumulation that is generally known in mammalian cells. Unlike human cells, Sfp53 accumulation and/or nuclear translocation could be observed selectively at the sublethal doses only. However, the possibility of high dose induced Sfp53 protein degradation as a cause of its reduced expression cannot be completely ruled out at this stage. In addition, maximal accumulation of Sfp53 following 200 Gy dose failed to alter the expression of some well-known p53 transcriptional targets (Fig. 2a,b). In sharp contrast, the ectopic overexpression of Sfp53 resulted in significant induction of cell death (Fig. 4a–c) which is in agreement with a previous study^25^. The unusual failure of radiation-accumulated Sfp53 to induce apoptosis (Fig. 1) hence suggests a radiation-induced selective inactivation, which we hypothesized to be due to a distinct functional conformation as compared to the ectopically overexpressed Sfp53. Since post-translational modifications could potentially cause such functional aberrations, we further studied post-irradiation p53 phosphorylation status. Quite importantly, the 200 Gy dose caused considerable hyper-phosphorylation that was completely absent in the untreated cells or cells irradiated at lethal doses (Fig. 5a). These findings thus show an unusual radiation response of a very well-conserved p53 orthologue contributing significantly to the radioprotection of lepidopteran cells. Certain *in vitro* studies with mammalian cell lines have also shown that p53 mutations/inactivation could be associated with enhanced radioresistance^3,4^, although radioprotective effect of its hyper-phosphorylation has not been known.

The p53-mediated regulation of cell cycle progression is one of the major cellular responses known to a variety of stressors including ionizing radiation. While p53-dependent cell cycle arrest (G1/S arrest induced primarily via p21 upregulation) may facilitate DNA repair, p53 activity may also lead to apoptosis depending upon the nature and extent (or irreparability) of damage. Irradiating Sf9 cells with different doses of radiation caused cell cycle arrest at G2/M phase. In Sf9 cells significant overexpression/- induction of p21 could not be distinctly observed, as with some other known p53 transcriptional targets (Fig. 2a,b), which may ultimately result in the failure of G1/S cell cycle arrest. Bae *et al*. have also suggested the failure of p53 to regulate the expression of p21 and suggest that Sf9 cells harbor a mutant allele of p53-like gene^26^. Additionally, dominant G2/M cell cycle arrest at sublethal doses (as also observed in Sf9 cells; Fig. 2b), could be a characteristic of null or mutated p53^37,38^. In agreement with our findings, previous studies have reported that silencing Sfp53 did not affect apoptosis stimulated by UV or camptothecin treatment. This raises the possibility that lepidopteran system may have undergone genetic alterations that make it less dependent on Sfp53 for apoptosis^27^. However, the possibility that p53 may transactivate pro-survival genes, and help in cell survival, cannot be ruled out.

In response to radiation, mammalian p53 is known to be regulated spatially and temporally by many post-translational modifications that occur at the amino- and carboxy-termini^39–41^. Acetylation is an important post-translational covalent modification of p53 observed in response to DNA damage^39,42^. The level of p53 acetylation may be an important regulator of p53 function, since p53 deacetylated by overexpressing histone deacetylase (HDAC) -associated proteins was shown to compromised in its ability to induce cell cycle arrest and apoptosis^43^. High acetylation of p53 was predicted in Sf9 cells, which is again well corroborated with considerably high HDAC activity, indicating its radiation-induced inactivation^36^.

Another major modification that occurs with p53 is its phosphorylation which helps in its accumulation and activation (Fig. 3g). The phosphorylation of p53 may occur in its amino terminus, leading to stabilization of p53 or in the carboxyl terminus and stimulating DNA binding^44–47^. The carboxy terminus of p53 can function as an autonomous domain capable of binding to different forms of DNA, such as damaged DNA, and reannealing complementary single strands of DNA or RNA. The hyper-phosphorylation of C-terminus of p53 was also shown to alter its DNA binding ability and cause the transcriptional inactivation of p53^34,35^. Sfp53 was also predicted to have a higher phosphorylation score of its C-terminus than human p53 and this could be a possible explanation for its altered transcriptional activity. This prediction was again supported by Sfp53 immunoprecipitation followed by western blot analysis using phospho-serine/phospho-threonine antibodies, which showed higher total phosphorylation of Sfp53 induced selectively at 200 Gy (Fig. 5a). That the hyper-phosphorylation of Sfp53 indeed contributes to protection of Sf9 cells was confirmed by its ability to significantly prevent Sf9 apoptosis induced by ectopic overexpression or lethal radiation doses. Firstly, Sf9 cells were transfected with full-length Sfp53 followed 16–18 h later by irradiation at 200 Gy, which invariably results in hyper-phosphorylation of p53 (Fig. 5a). Secondly, Sf9 cells were first primed with sublethal dose (200 Gy) and then irradiated with lethal dose (2000 Gy) 24 h later. In both cases, irradiation at 200 Gy resulted in significant inhibition of apoptosis (Fig. 5b,c). These findings thus demonstrate a strong cyto-protective role of phosphorylated Sfp53.

We noticed a reverse pattern in the radiation dose-response of p53 versus miR-31 (Fig. 1). The miR-31 expression was selectively suppressed at 200 Gy dose, which coincidentally resulted in the maximum accumulation of p53. Recently, we had reported that miR-31 overexpression mediates radiation-induced apoptosis in these model insect cells^19^. Therefore, subdued miR-31 levels at 200 Gy could be the result of an intrinsic radioprotective mechanism, and indicated a possible relation with Sfp53 response. Quite importantly, the knockdown of Sfp53 prior to irradiation at 200 Gy released the repression of miR-31 and induced significant cell death; thus demonstrating the existence of a ‘p53 gateway’ with a strong radioprotective role in these cells (Fig. 7). It should be noted here that the absence of accumulation and/or hyper-phosphorylation of Sfp53 following irradiation at the lethal doses (1000Gy-3000Gy) was associated with significant dose-dependent induction of cell death (Figs 1a, 5a), which also involved dose-dependent miR-31 overexpression^19^. The radiation-induced post-translational changes in Sfp53 resulted in altered DNA binding ability. Our study shows differential binding of Sfp53 at the miR-31 locus selectively following irradiation at 200 Gy (Fig. 6b), which strongly suggests a direct transcriptional repression of miR-31 by hyper-phosporylated Sfp53. However, the exact Sfp53 binding site at miR-31 locus in Sf9 is not possible to detect because of the unavailability of complete annotated lepidopteran genome sequence at present. A possible explanation for p53-mediated miR-31 suppression could be that phosphorylated p53 may bind at the miR-31 locus and may negatively compete with other transcriptional factors required for inducing expression of miR-31. The higher (lethal) radiation doses failed to display similar response as Sfp53 accumulation was diminished in a dose-dependent manner, which corresponded very well with the overexpression of miR-31. Although upstream mechanisms facilitating a dose-selective p53 accumulation would need further investigations, the present study nevertheless reveals that the radioresistance of these model insect cells is significantly aided by a miR-31 repressing and ‘radioprotective p53 gateway’. Since Sfp53 has abundant homology with the human p53, this study may have significant implications for modulating cellular radioresistance.

## Materials and Methods

### Cell line and irradiation

Sf9 cell line, (originally established from the ovaries of *Spodoptera frugiperda*) obtained from National Institute of Immunology, New Delhi, India, was maintained as semi-adherent monolayer in the 25 cm^2^ culture flasks (Falcon, BD Biosciences, USA) at 28 °C in Grace’s insect cell medium (Sigma, St Louis, MO, USA) supplemented with 3.33 g/l lactalbumin hydrolysate (Sigma), 3.33 g/l yeastolate (BD Biosciences, San Jose, CA, USA), 0.35 g/l NaHCO3 and antibiotics (Penicillin sodium salt 50,000 U/l, streptomycin sulfate 50,000 μg/l, Nystatin 2000 μg/l from 500,000 USP U/mg; Sigma). Growth medium (pH 6.2) was prepared by adding 10% heat inactivated Fetal Bovine Serum (FBS) (Sigma, USA) and stored at 2–8 °C. Cells were regularly subcultured twice a week in exponential phase and were seeded at 10^6^ cells/flask (35,000–40,000 cells cm^−2^) in 5 ml of growth medium. Irradiation was carried out in the exponential phase of cell growth at a dose rate of 19.16 Gy min^−1^ in Gamma Chamber-5000 (Board of Radiation and Isotope Technology, Department of Atomic Energy, Mumbai, India). Based on our previous studies^19,24,48,49^ 24 h post-irradiation time point was selected (unless specified otherwise) for cell death and protein expression analysis, since Sf9 cells undergo substantial cell death primarily at 24h-48h following irradiation at high doses.

### RNA/miRNA expression analysis

Total RNA and miRNA isolation was performed using RNeasy kit (Qiagen, USA) and mirVana miRNA Isolation Kit (Ambion, USA) respectively as per manufacturer recommendations. 1 μg of total RNA and miRNA was used for cDNA synthesis in 20 μl reaction using Verso Reverse Transcriptase (Thermo Scientific, USA) and miScript Reverse Transcription Kit (Qiagen, Valencia, CA, USA) respectively as per manufacturer’s protocol. 2 μl of cDNA was used to analyze the mRNA/miR-31 expression using gene specific primers in Real Time Thermocycler (Stratagene, USA, MX3005P). Single peak of dissociation curve from the amplified produced was considered as specific amplification.

### Immuno-precipitation and western blot analysis

For protein expression analysis, western immunoblotting was performed at different time points after irradiation, as detailed earlier^50^. For p53 immunoprecipitation cells were irradiated with sub-lethal and lethal doses and harvested 24 h post-irradiation. Cells were lysed using native lysis buffer (150 mM NaCl, 10 mM HEPES [pH 7.4], 1% CHAPS, 10% Protease inhibitor cocktail) and 250 μg of whole cell lysate was used for immunoprecipitation using Catch and Release kit (Millipore, USA) as per manufacturer’s protocol. Eluted proteins were separated on 15% PAGE and transferred onto PVDF membrane. Detection of phosphorylated Sfp53 was performed using phosphor- Serine, Threonine specific antibodies and Enhanced Chemiluminescence (ECL) reagent (Pierce, USA). The position of phospho-Sfp53 was marked by p53 specific antibody on the same membrane.

### Cloning of Sfp53

Sfp53 nucleotide sequence was extracted from NCBI (Accession: HM773026.1, GI: 329755764). To clone Sfp53 total RNA was isolated from exponentially growing Sf9 cells using RNeasy mini Kit (Qiagen) and 5 μg of total RNA was converted to cDNA with Verso cDNA Kit (Thermo Scientific, USA) as per manufacturer’s instructions. For error free amplification of full length p53, AccuPrime Pfx DNA polymerase (Invitrogen, USA) was used with 0.1μM final concentration of specific primers. The sequence of the primers is:

Forward Primer- CACCATGGAGCATTATGATATGCCGTAT

Reverse Primer- CTGTGACAACGGCGGCGG.

After PCR amplification the amplified products were resolved on a 1% agarose gel. Single band of the amplified product (size ~1200 bp) represented as specific amplification was gel purified using QIAquick Gel extraction kit (Cat. No. 28704, Qiagen) and was cloned in pcDNA3.2 vector using PcDNA/V5/GU/D-TOPO expression Kit (Invitrogen, USA, Cat No. K2440-20,) as per manufacturer’s recommendation. The orientation and sequence of the cloned Sfp53 was confirmed by sequencing with flanking T7 and TK primers.

### SiRNA and Sfp53 clone transfection

For transfection of AS-miR-31 and siRNA against p53, 0.50 μg of each was used with RNAiFect (Qiagen, USA, Cat No. 301605) in 1:5 ratio (w/v). The expression of miR-31 after antisense transfection was quantified using Real Time PCR after 16–18 h, while p53 expression analysis was performed 24 h post transfection with western blotting. For Sfp53 clone transfection 1 μg of clone was used with 6 μl of superFect transfection reagent (Qiagen, USA). Overexpression of Sfp53 was assessed by western blotting after 24 and 48 h after transfection. All the transfections were carried out in exponential phase of cell growth.

### Cell death Measurement

Cell death was assessed by observing cells under DIC microscope (Axiovert-200 Zeiss inverted DIC microscope, Carl Zeiss, Germany). Caspase-3 activity assay and cell morphology assay^51^ were performed to determine dead cell population. Caspase-3 activity was assessed 24 h post-irradiation using caspase-3 activation assay kit (Sigma, Cat No. CASP3F) according to manufacturer’s recommendation. The mean fluorescence value of triplicate samples were plotted to determine variation in caspase-3 activation. For cell morphology assay, cells were harvested 24 h following irradiation with or without transfection with AS-miR-31. Direct cell suspension was used for embedding on agarose-laden slides and fluorescence staining with FITC-PI for assessing morphological changes.

### Cell cycle analysis

For cell cycle analysis by flow cytometry, cells were harvested 24 h post irradiation with or without pre-treatment by siRNA. Cells were then washed with PBS followed by fixation in 70% ethanol and kept for overnight incubation at −20 °C. Propidium iodide (20 μg/ml) was used for nuclear staining following RNase treatment for 30 min at 37 °C. Relative DNA content and cell cycle distribution was analyzed with FACSCalibur flow cytometer (Becton- Dickinson & Co., New Jersey, USA) using the CellQuest software (v.3.0.1) for acquisition and ModFit LT software (v.2.0; Verity Software House, Inc., Maine, USA) for cell cycle analysis.

### *In silico* analysis

Primary sequence of Spodoptera p53 (AEC04309.1) was extracted from NCBI database and used to predict molecular structure using I-TASSER online platform. Characterization of the different domains of Sfp53 was performed on the basis of sequence similarity search using BioEdit software. The interaction of p53 with different proteins was observed by Hex 8.0 software. The p53 N-terminus interaction with TAF9 and p53 dimerization capacity was studied using Hex 8.0 tool using Sfp53 model. The phosphorylation status of Sfp53 was predicted by NetPhos 2.0^52^ and GPS 2.1^53^. Acetylation status of the Carboxy terminus domain (CTD) was predicted using PAIL: Prediction of Acetylation on Internal Lysines^54^ and LAceP: Lysine Acetylation Site Prediction^55^.

### Chromatin Immunoprecipitation (ChIP) assay and Dot blot analysis

Cells were irradiated with 200 Gy and 2000Gy and cross-linked using 3.5% final concentration of formaldehyde for 20 min. at room temperature. The cross-linking was terminated by the addition of glycine to a final concentration of 125 mM for 5 min and cells were collected by centrifugation at 2000 rpm for 5 min followed by a 1X PBS wash. Cell pellet was resuspended in RIPA cell lysis buffer (10 mM Tris pH 7.6/1 mM EDTA/0.1% SDS/0.1% sodium deoxycholate/1% Triton X, with 1X protease inhibitor cocktail) for 10 min followed by sonication on ice (using a Bandelin Sonopuls sonicator) with a micro tip in 15-sec bursts followed by 30 s of cooling on ice for a total of 40 cycles at 100% power setting to achieve chromatin fragments of about 1000 bp–2000 bp. Cell debris was cleared by centrifugation at maximum speed for 10 min at 4 °C and sample was pre cleared with 50 µL of protein G plus/A agarose slurry (calbiochem) for 30 min at 4 °C on a rotating disk. The purified samples were three fold diluted in (1:3) ChIP dilution buffer (0.01% SDS/1.1% Triton X-100/1.2 mM EDTA, 16.7 mM Tris, pH 8.1/167 mM NaCl and protease inhibitors) along with 60 µL of protein G plus/A agarose slurry and incubated overnight at 4 °C with rotation. Beads were then washed consecutively for 2–3 minutes on a rotating disk with 1 mL of each of the following solutions: **a**. Low salt wash buffer [0.1% SDS/1% Triton X-100/2 mM EDTA, 20 mM Tris, pH 8.1/150 mM NaCl] **b**. High salt wash buffer [0.1% SDS/1% Triton X-100, 2 mM EDTA, 20 mM Tris, pH 8.1, 500 mM NaCl] **c**. LiCl wash buffer [0.25 M LiCl/1% NP40/1% deoxycholate, 1 mM EDTA/10 mM Tris, pH 8.0] **d**. Twice in 1X TE buffer

Finally elution was carried out in 100 µL of elution buffer (1% SDS/0.1 M NaHCO) from pelleted beads. After elution, samples were decrosslinked by adding 1 µL of RNase (10 mg/ml) and 5 M NaCl to a final concentration of 0.3 M to the elution buffer and incubated at 70 C° for 5–6 hours. Samples are then precipitated overnight by adding 3 times the volume of 100% ethanol at −20 °C followed by centrifugation at maximum speed at 4 °C. DNA pellet was resuspended in 100 μl of sterile water. To reduce protein contamination 2 µL of 0.5 M EDTA, 4 µL 1 M Tris, pH 6.5 and 2 µL of 8 mg/ml Proteinase K was added to the elute and incubated for 1–2 hours at 45 °C. DNA was purified using Qiagen Mini elute spin columns and eluted in 50 µL/column of elution buffer. Precipitated DNA was subsequently used for Dot blot to verify whether miR-31 also gets immunoprecipitated with p53.

To detect p53 binding at miR-31 locus, DNA isolated from p53 immunoprecipitated samples at different radiation doses (200 Gy and 2000 Gy) were used. Nylon membrane was pretreated with 2X SSC buffer (30 mM sodium citrate/pH 7.0/0.3 M NaCl) for 5 min. 12 μl of individual ChIP samples were diluted with 2X hybridization buffer (mirVana, miRNA detection kit, Ambion) and then boiled for 10 min followed by addition of equal volume of 1 M NaOH and incubated at room temperature for 20 min. The samples were then spotted on the pretreated nylon membrane using Bio-Dot apparatus (BIO-RAD, Cat No. 1706545) and vacuum apparatus (BIO-RAD, Cat. No.1706545). Membrane was then subjected to UV crosslinking at 254 nm for 10 min and incubated in a neutralizing solution (I mM EDTA/1.5 M NaCl/0.5 M Tris/pH 7.2) for 30 min at room temperature. Membrane was rinsed in 2X SSC buffer and air dried before hybridization. For biotinylated probe hybridization, 5 μl of probe was used for each sample. Membrane was then incubated at 42 °C for 45 min. and washed with washing solution (Cat No. 89880, Pierce Nucleic acid chemiluminescent detection kit) to remove any unhybridized residue. The membrane was blocked with blocking buffer provided with the kit (Chemiluminescent Nucleic acid detection module, Thermo scientific) and subsequently incubated with a streptavidin HRP conjugate for 30 min and washed thrice before being developed on X-ray film.

### Immuno-fluorescence microscopy

To study p53 translocation, cells were grown on cover slips and irradiated with different doses of γ- radiation with and without siRNA and incubated for 16–20 h at 28 °C. Following irradiation, cells were washed with wash buffer (PBST with 0.01% bovine serum albumin (BSA)) and fixed with 2% para-formaldehyde in PBST for overnight at 4 °C. Permeablization was carried out using permeabilization buffer (PBS with 0.5% Saponin, 0.05% TritonX100; Sigma, USA) for 10 min at 4 °C. The cells were again washed with cold wash buffer and incubated with mouse monoclonal antibody pAb 1801 to p53 N-terminus (Santa Cruz, dilution 5 μg/10^6^ cells) for 1 h, followed by incubation with FITC-conjugated secondary antibody (Sigma, USA) for 45 min in the dark. Both the antibodies were diluted in antibody dilution solution (PBS containing 0.01% saponin, 1% BSA, 0.1% sodium azide; Sigma, USA). After thorough washings, coverslips with labeled cells were mounted on microscopic slides and images were taken using DIC-fluorescence microscope (Axiovert 200, Carl Zeiss, Germany) and Axiovision software (version 4.0).

### Detection of p53 phosphorylation

Cells were irradiated with sublethal (200 Gy) and lethal doses (2000 Gy) and harvested 24 h post-irradiation, washed with PBS and resuspended in native lysis buffer (HEPES 45 mM, KCl 55 mM, TritonX-100 1.1%, EDTA 5 mM, Sodium orthovanadate 1 mM, sodium fluoride 50 mM, phenylmethylsulfonyl fluoride 1 mM, Benzamidine 1 mM, Protease inhibitor 10%) for 1 h. Lysate was then used for immunoprecipitation using Catch and Release kit as per manufacturer’s recommendations. Anti-p53 antibody (Santa Cruz, USA, PAB-1801) was used for overnight precipitation of Sfp53 at 4 °C. Protein was recovered after elution and transferred to PVDF membrane after its separation by SDS-PAGE. Phospho-serine and phosphor-threonine antibodies (both from Qiagen) were used to detect phosphorylation of immunoprecipitated p53. To mark the exact position of Sfp53 on membrane p53 specific antibodies and molecular weight marker was used.

### Statistical analysis

Each experiment was repeated at least three times with triplicate samples. Mean values and standard deviations were calculated and plotted. Student’s *t* test (unpaired) was used to examine the statistical significance of differences between groups, wherever applicable.

### Data availability

All data generated or analyzed during this study are included in this published article.
